# Hospital Meetings, &c.

**Published:** 1899-06-03

**Authors:** 


### HOSPITAL MEETINGS, &C.

### HOSPITAL FOR CONSUMPTION, BROMPTON.

The annual Court of Governors of this hospital was held in the board hospital room on Thursday, the 25th inst. The Right Hon. the Earl of Derby, K.G., G.C.B., president of the corporation, presiding. The Vice-President, Mr. T. P. Beckwith, Colonel the Lord Arthur Wellesley, the Hon. Dudley Fortescue, and Mr. J. G. Noel, C.B., were among the governors who attended. The fifty-eighth annual report of the managing committee was read by the secretary, Mr. W. H. Theobald. It stated that the receipts for the year totalled £34,655, compared with £45,000 in the previous year. Annual subscriptions, which were in 1897 £7,660, amounted in 1898 to £7,669, and the donations were £4,200 as against £2,824 in the preceding year. £13,098 had been received from legacies, as against £22,132. The in-patients numbered 1,804, the out-patients 13,988, as compared with 13,098 in 1897, and the attendances were 74,338, an increase of nearly 3,000 on the total number of the previous year. It was announced that Mrs. Ellen Price would be elected as Lady Superintendent in the place of Miss C. Davidson, who had resigned. The "Country Branch and Convalescent Home," the memorial of her Majesty's Diamond Jubilee, is making progress towards establishment. A sub-committee has been appointed to select a convenient site. Funds, says the report, are urgently needed both for this and for the home for private and staff-nurses in South Parade, which is now nearing completion. Three memorial wards had been named during the year, the "Caroline Anna Fletcher," the "Richard Tibbs," and the "Helen Faucit," the last by Sir Theodore Martin, K.C.B., in memory of his wife, Lady Martin.

In moving the adoption of the report, the Chairman mentioned the loss the committee had sustained in the deaths of three of its members—Colonel the Hon. Sir W. Patrick Talbot, K.C.B., Mr. Henry Rose, and Mr. Charles Cecil Cotes. They also deeply regretted the death of Dr. C. J. Arkie, one of the most promising members of the medical staff, whose professional ability was of a high order. The Chairman proceeded to refer with regret to the resignation of the Duke of Norfolk, which his Grace has been asked to reconsider. One prominent and very satisfactory point in the report was the evidence given by the large increase in the out patient department of the great benefits it conferred. The falling-off in subscriptions since 1896 must be regarded as merely accidental, and not as indicating any decrease of interest in the hospital on the part of the public. That the public estimation remained the same ample proof was given in the large number of legacies received during the year. In the donations there was an increase of £1,376 over those of 1897. The fact of the expenditure having slightly risen while there was a formidable decrease in the receipts was of no great moment, that being a state of affairs which occasionally recurred, and which was counterbalanced by those years which showed a difference on the right side. The giving of large donations seemed to form a reason for the abstinence from subscriptions, but, the noble Earl remarked, the hospital was by no means disdainful of the annual subscriptions. The giving of these conferred on the hospital not only a monetary benefit, but also extended public interest in its welfare by bringing more people into contact with it. In referring to the many sources of expenditure that the committee had before them this year, his lordship remarked that if the public were heartily interested in the establishment of the country branch, which would make large demands upon the resources of the hospital, they must show their readiness to help. The fresh-air treatment was to be tried, as far as circumstances would allow, in the south portion of the building; but it was eminently desirable to have some place out of London where the treatment might be carried on under more favourable conditions. In conclusion the noble chairman remarked that as regards consumption, of all the countries in which it is prevalent, England has the lowest rate of mortality.

The report having been adopted, a committee of management was then appointed, and a vote of thanks offered to the medical staff for their valuable services. The Vice-President (Mr. T. P. Beckwith), in seconding the vote of thanks to the noble chairman, referred to Lord Derby's notice of the satisfactory increase in the out-patients' department. This was a real, and not apparent benefit, for he was happy to say the hospital had not suffered from the illegitimate use of its privileges by outsiders.

The Earl of Derby, in responding to the vote of thanks, said that, remembering the difficulty there was at one time to get people to attend the hospitals, he was inclined to look leniently upon those who received from them benefits to which they had no just claim.

The proceedings then terminated.

### MIDDLESEX HOSPITAL.

The Right Hon. Sir Ralph Thompson, K.C.B., presided at a well-attended meeting of the quarterly court of governors of the Middlesex Hospital held May 25th. It was announced that arrangements were now in progress for the accommodation of patients suffering from tuberculous disease of the lungs at the convalescent home at Clacton-on-Sea, where the board had set aside a certain number of beds in order to try experimentally the "open air" treatment for this disease. There had been a falling off in the amount of annual subscriptions for the past quarter, and an earnest appeal was made for additional support. 1,069 patients had been admitted during the quarter, of whom 625 had been discharged cured, 213 of this number being sent to the convalescent home, whilst at the end of the quarter 294 still remained in the hospital. In the out-patient department 11,443 had been seen, and these had attended a total of 28,477 times. The new wing for female cancer patients will shortly be opened.

### BRITISH HOME FOR INCURABLES.

#### Laying the Foundation-stone of New Wing.

On May 30th the board of management gave a garden-party on the occasion of Earl Amherst's laying the foundation-stone of the new wing of the British Home for Incurables, Crown Lane, Streatham. After singing the National Anthem, the chaplain repeated a few short prayers, and the president (Lord Amherst), at the request of the chairman (Mr. Bevan), after due preparation, declared the stone "well and truly laid." Mr. C. Ernest Tritton, M.P., and Colonel Campbell, L.C.C., then addressed the assembly. An urgent appeal was put forth for the sum of £12,000 to cover the expenditure on the proposed building and to endow the beds. The new wing was contemplated in the original plans of the home, of which the foundation-stone was laid in 1894 by the Princess of Wales, and the further accommodation was now much needed, both for the reception of eligible patients waiting to come in and also for the comfort of the staff and for a recreation room. The Princess of Wales approved the proposed extension of the home, and the appeal for funds to endow the new beds had received the sanction and support of Her Royal Highness. Lord Amherst's speech was very short. He thanked the people for their presence, complimented the Board of Management and the secretary on the excellence of the arrangements, and advised everyone to go into the garden and enjoy the entertainments provided and to give a good donation to the funds of the home. A collection realising £60 was made during the singing of the final hymn. In the course of the afternoon a telegram was received from the Princess of Wales expressing her sympathy with the efforts being made to increase the usefulness of the home, and thanking Lord Amherst for laying the foundation-stone. The day was fine, the entertainments varied and excellent, and the 300 guests appeared to enjoy themselves thoroughly. The home was open to inspection, and as many of the inmates as possible were out of doors taking part in the proceedings.

